# Increased Systemic Immune-Inflammation Index Is Associated With Delayed Cerebral Ischemia in Aneurysmal Subarachnoid Hemorrhage Patients

**DOI:** 10.3389/fneur.2021.745175

**Published:** 2021-10-11

**Authors:** Liuwei Chen, Sajan Pandey, Rui Shen, Yi Xu, Quanbin Zhang

**Affiliations:** Department of Neurosurgery, Shanghai Tenth People's Hospital, Tongji University School of Medicine, Shanghai, China

**Keywords:** systemic immune-inflammation index, delayed cerebral ischemia, inflammation, aneurysmal subarachnoid hemorrhage, hemorrhagic stroke

## Abstract

**Background:** Systemic immune-inflammation index (SII) is a novel biomarker that reflects the state of a patient's inflammatory and immune status. This study aimed to determine the clinical significance of SII as a predictor of delayed cerebral ischemia (DCI) in patients with aneurysmal subarachnoid hemorrhage (SAH).

**Methods:** Retrospective data were collected from aneurysmal SAH patients who had been admitted to our hospital between January 2015 and October 2019. Both univariate and multivariate analyses were performed to investigate whether SII was an independent predictor of DCI. In addition, the receiver operating characteristic (ROC) curve and area under the curve (AUC) were also evaluated.

**Results:** There were 333 patients with aneurysmal SAH included in this study. Multivariate logistic analysis revealed that a modified Fisher grade 3 and 4 score [odds ratio (OR) = 7.851, 95% confidence interval (CI): 2.312–26.661, *P* = 0.001] and elevated SII (OR = 1.001, 95% CI: 1.001–1.002, *P* < 0.001) were independent risk factors for DCI. ROC curves showed that SII could predict DCI with an AUC of 0.860 (95% CI: 0.818–0.896, *P* < 0.001). The optimal cut-off value for SII to predict DCI was 1,424, and an SII ≥ 1,424 could predict DCI with a sensitivity of 93.1% and a specificity of 68.1%. Patients with higher SII value on admission tended to have higher incidence of acute hydrocephalus and DCI, greater modified Fisher and Hunt-Hess scales, and poorer outcomes.

**Conclusions:** SII is an independent predictor of DCI in patients with aneurysmal SAH. The SII system can be implemented in a routine clinical setting to help clinicians diagnose patients with high risk of DCI.

## Introduction

Aneurysmal subarachnoid hemorrhage (SAH) is a critical cerebrovascular accident with high financial and disease (mortality and morbidity) burdens (1, 2). The mechanisms of cerebral injury following aneurysmal SAH remain largely unclear. However, it is believed that the risks of inflammation and thrombosis may be increased after aneurysmal SAH (3, 4).

Delayed cerebral ischemia (DCI) is often associated with poor functional outcomes in patients who survived the first-time SAH (5, 6). DCI can lead to poorer prognostic outcomes, greater disease severity, and higher mortality rates in patients with aneurysmal SAH (7). The pathophysiological characteristics of DCI include cerebral inflammation, micro-thrombosis, perfusion mismatch, spreading depolarization, and neurovascular uncoupling, all of which culminate in an infarction event (8). Therefore, identifying aneurysmal SAH patients who are at high risk of DCI can improve the survival outcomes of these patients.

Leukocytosis and platelet activation occur after aneurysmal SAH due to the stimulation of systemic immune reaction (9). Elevated inflammatory biomarkers, such as neutrophil-lymphocyte ratio (NLR) and platelet-lymphocyte ratio (PLR), have been employed to predict DCI development and functional outcome following aneurysmal SAH (10). Systemic immune-inflammation index (SII) is a novel biomarker reflecting the balance of inflammatory and immune status, which can be calculated as platelet count × neutrophil count/lymphocyte count. SII has recently been shown to exhibit a good prognostic value in predicting different malignant conditions (11–13). However, the ability of SII to predict DCI remains unclear. Therefore, this study aimed to evaluate the potential of SII as a predictive biomarker of DCI and to compare its effectiveness with other inflammatory biomarkers.

## Methods

### Patients

We retrospectively collected and analyzed data from aneurysmal SAH patients who received treatment in our hospital between January 2015 to October 2019. The patients were included if they met the following criteria: (i) aneurysmal SAH confirmed by CT angiography and/or digital subtraction angiography, (ii) endovascular coiling or surgical clipping within 48 h following admission, and (iii) <24 h between symptom onset and hospital admission. The patients were excluded if they had (i) additional cerebral vascular disease (e.g., arteriovenous malformation or moyamoya disease), (ii) prior severe systemic disease (e.g., renal, hepatic, immune, hematologic, and/or infectious diseases) or history of malignancy, (iii) death within 3 days of admission, and (iv) missing data. Ethical approval for this study was obtained from the institutional ethics review board.

### Demographical, Clinical, and Laboratory Parameters

The collected data included age, gender, medical history, radiological characteristics (modified Fisher grade, acute hydrocephalus), clinical status at admission (Hunt-Hess grade), aneurysm location and size, treatment approach (coiling or clipping), and DCI occurrence. Acute hydrocephalus was characterized as the presence of hydrocephalus symptoms and ventricle enlargement (bicaudate index ≥ 0.20 on CT scan) within 72 h following aneurysmal SAH. According to the AHA/ASA (14), DCI was defined as new focal neurological impairment or a decrease of two points on the Glasgow Coma Scale, which did not appear immediately following aneurysm occlusion. The management of SAH patients and DCI was carried out according to the SAH management guideline (15, 16). In sedated or poor-grade SAH patients, transcranial doppler ultrasonography is routinely used to detect vasospasm and DCI (16). Functional outcomes were evaluated using the modified Rankin Scale (mRS) score at 3 months. Data were obtained by telephone or outpatient interview. For analysis purposes, the data were dichotomized as follows: modified Fisher scale (“1–2” and “3–4”), Hunt-Hess scale (“1–3” and “4–5”), aneurysm location (“anterior cerebral artery,” “internal cerebral artery,” “middle cerebral artery,” and “vertebrobasilar artery”), aneurysm size (“>10 mm,” “5–10 mm,” and “<5 mm”), treatment approach (“clipping” and “coiling”), and functional outcome (“>2” and “≤ 2”). Blood specimens were collected before treatment at admission and analyzed by an autoanalyzer (XE-2100, Sysmex Company, Japan) within 1 h after venipuncture. SII was calculated as platelet count × neutrophil count/lymphocyte count; NLR was calculated as neutrophil count/lymphocyte count, and PLR was calculated as platelet count/lymphocyte count.

### Statistical Analysis

All statistical tests were conducted with SPSS v21.0 and Medcalc v19.1. Continuous variables were expressed as median (interquartile range, IQR), while categorical variables were presented as numbers (percentages). Mann-Whitney U test was used to analyze the continuous univariate variables, whereas χ^2^-test or Fisher exact-test was employed to assess the categorical univariate variables. Potential predictors of DCI were examined through analysis of multicollinearity by stepwise regression. Significant univariate variables in univariate logistic analysis (*P* < 0.10) were inputted into a multivariate regression model. Receiver operating characteristic (ROC) curves were constructed to determine the discriminative abilities of SII, NLR, and PLR for predicting DCI. Area under the curve (AUC) was subsequently evaluated. The ROC curves were also employed to establish the optimal cut-off point at which the sum of the sensitivity and specificity values was the highest. The discriminative performances of the NLR, PLR, and SII were compared using the DeLong test. A two-sided *P-*value of < 0.05 was deemed statistically significant.

## Results

**Figure 1:**
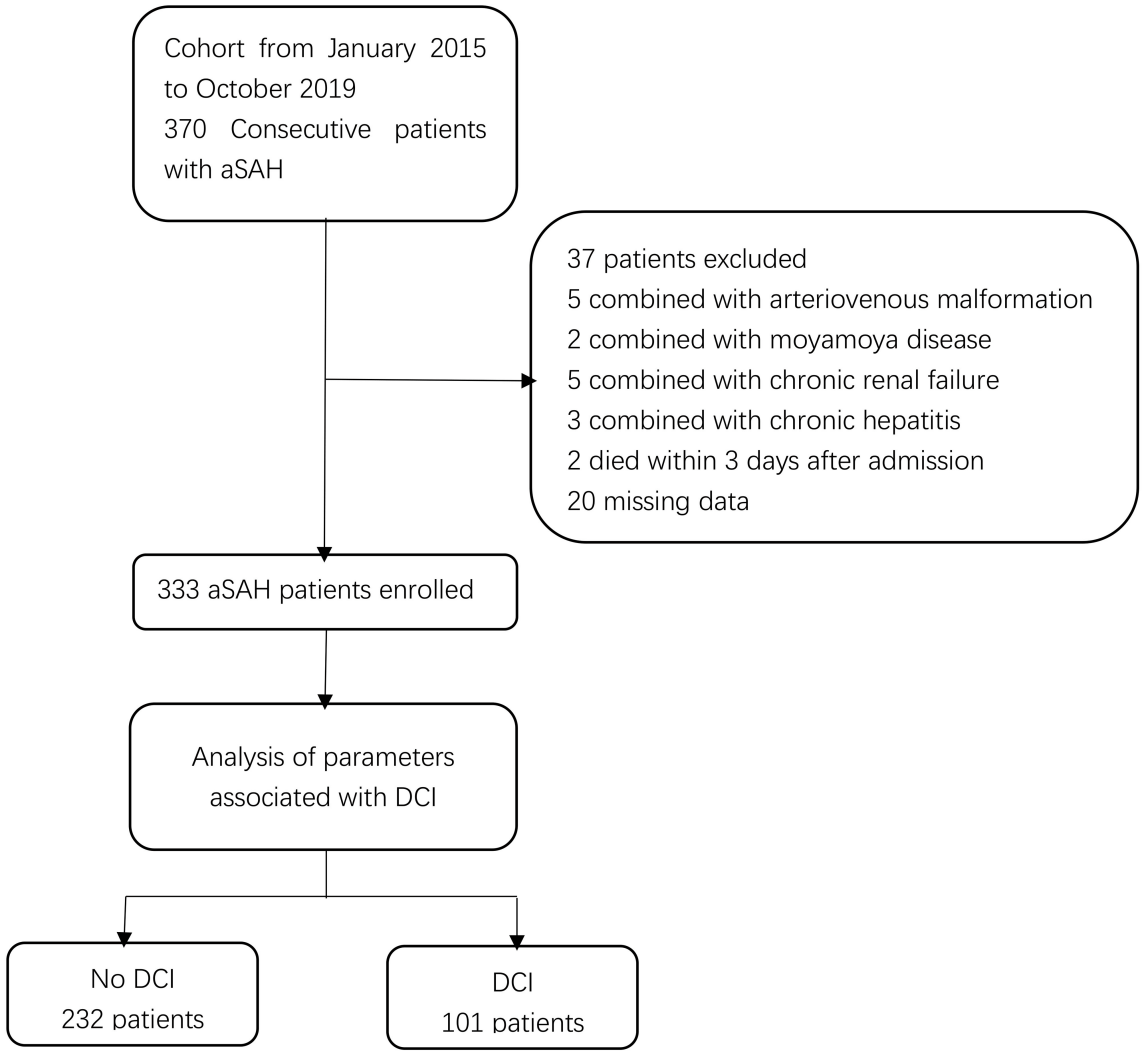
Flow chart of study patients. aSAH, aneurysmal subarachnoid hemorrhage; DCI, delayed cerebral ischemia.

Three hundred thirty-three aneurysmal SAH patients, including 205 (61.6%) female and 128 (38.4%) male, were enrolled into this study (Figure 1). The median age at the time of admission was 59 years (range, 51–66). In addition, 81 (24.3%) and 252 (75.7%) patients received surgical clipping and endovascular coiling, respectively. Furthermore, DCI occurred in 101 (30.3%) patients.

**Figure 2:**
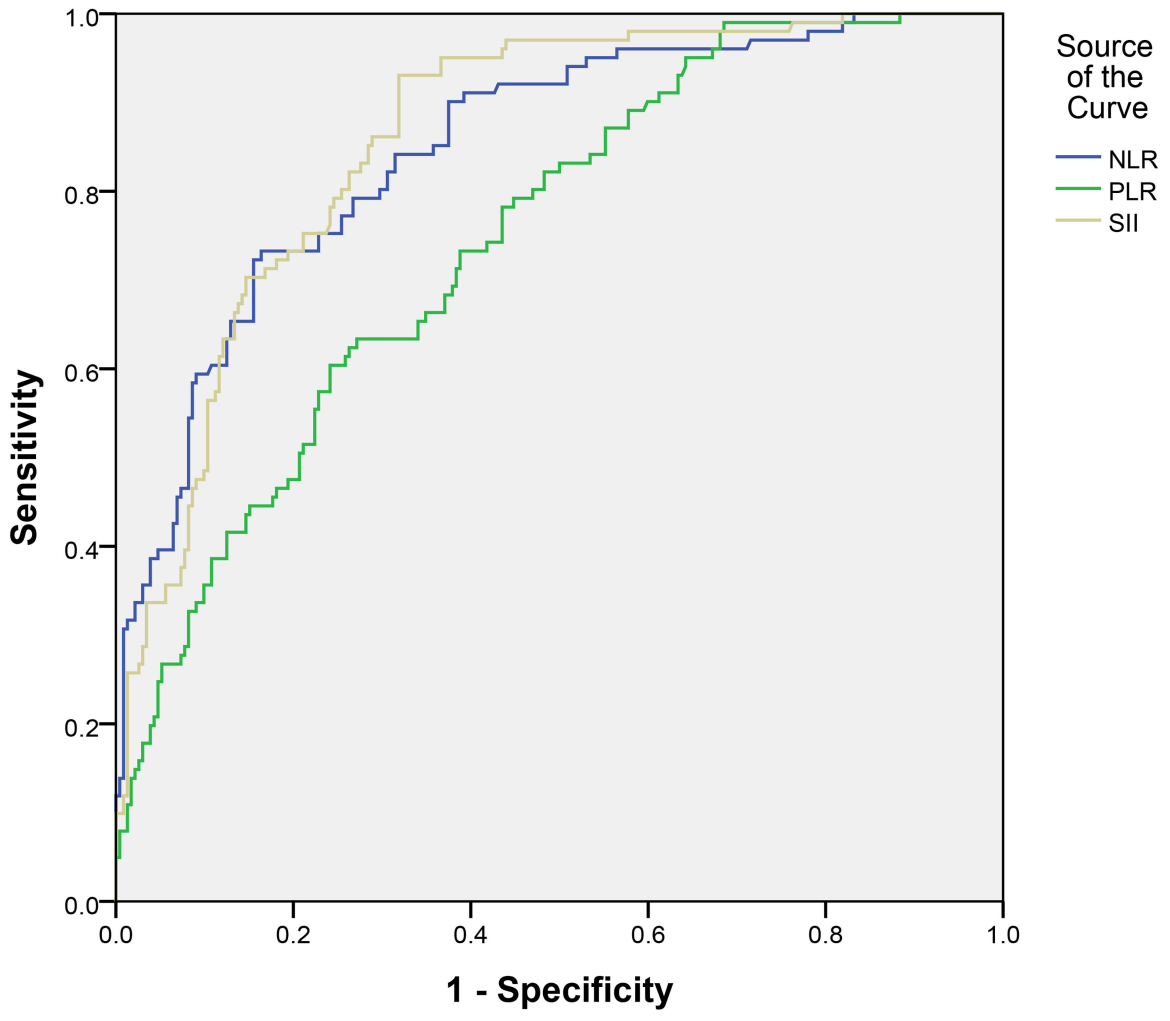
Discriminative ability of the SII, NLR, and PLR for DCI. SII, systemic immune-inflammation index; NLR, neutrophil-lymphocyte ratio; PLR, platelet-lymphocyte ratio.

All patients were assigned to two groups based on the occurrence of DCI. Univariate analysis revealed that aneurysmal SAH patients with DCI tended to have higher incidence of acute hydrocephalus, to have greater modified Fisher and Hunt-Hess scores, and to be treated by surgical clipping (Table 1). Moreover, the red blood cell count, hemoglobin, and lymphocyte percentage were remarkably lower in patients with DCI than those without DCI (Table 1). Meanwhile, the white blood cell count, c-reactive protein (CRP), and SII value were markedly higher (Table 1). Multivariate logistic regression analysis demonstrated that modified Fisher grade 3 and 4 score [odds ratio (OR) = 7.851, 95% confidence interval (CI): 2.312–26.661, *P* = 0.001] and SII (OR = 1.001, 95% CI: 1.001–1.002, *P* < 0.001) were independent risk factors for DCI development (Table 2). ROC curves showed that SII could predict DCI with an AUC of 0.860 (95% CI: 0.818–0.896, *P* < 0.001; Figure 2). The AUC for SII was obviously greater compared to PLR, but the difference was not significant between NLR and SII (Table 3). The optimal cut-off value for SII to predict DCI was 1,424, and an SII ≥ 1,424 could predict DCI with a sensitivity of 93.1% (95% CI: 86.2–97.2%), a specificity of 68.1% (95% CI: 61.7–74.1%), a positive predictive value of 56.0% (95% CI: 51.1–60.7%), and a negative predictive value of 95.8% (95% CI: 91.7–97.9%).

**Table 1:** Comparison of demographic, clinical, and laboratory data in patients with aneurysmal SAH according to the development of delayed cerebral ischemia.

**Variable**	**Total (*n* = 333)**	**DCI**		***P*** **-value**
		**Yes (*n* = 101)**	**No (*n* = 232)**	
Age (years)	59 (51–66)	60 (53–67)	59 (50–66)	0.288
Sex (female)	205 (61.6%)	56 (55.4%)	149 (64.2%)	0.130
Hypertension	189 (56.8%)	55 (54.5%)	134 (57.8%)	0.576
Diabetes	45 (13.5%)	13 (12.9%)	32 (13.8%)	0.821
Hunt-Hess grade				<0.001
Grade 1,2,3	235 (70.6%)	37 (36.6%)	198 (85.3%)	
Grade 4,5	98 (29.4%)	64 (63.4%)	34 (14.7%)	
Modified Fisher grade				<0.001
Grade 1,2	101 (30.3%)	4 (4.0%)	97 (41.8%)	
Grade 3,4	232 (69.7%)	97 (96.0%)	135 (58.2%)	
Location				0.118
ACA	115 (34.5%)	35 (34.7%)	80 (34.5%)	
ICA	123 (36.9%)	33 (32.7%)	90 (38.8%)	
MCA	50 (15.0%)	22 (21.8%)	28 (12.1%)	
VBA	45 (13.5%)	11 (10.9%)	34 (14.7%)	
Size (mm)				0.272
<5	193 (58.0%)	53 (52.5%)	140 (60.3%)	
5–10	114 (34.2%)	41 (40.6%)	73 (31.5%)	
>10	26 (7.8%)	7 (6.9%)	19 (8.2%)	
Treatment				0.001
Coiling	252 (75.7%)	64 (63.4%)	188 (81.0%)	
Clipping	81 (24.3%)	37 (36.6%)	44 (19.0%)	
Acute hydrocephalus	48 (14.4%)	31 (30.7%)	17 (7.3)	<0.001
RBC, ×10^9^	4.1 (3.6–4.4)	3.9 (3.2–4.4)	4.1 (3.7–4.4)	0.023
Hemoglobin	122 (110–135)	118 (100–134)	124 (114–135)	0.007
WBC, ×10^9^	9.7 (7.7–12.2)	11.9 (10.3–15.1)	8.6 (7.4–11.3)	<0.001
Neutrophil %	83.6 (78.0–87.8)	84.4 (78.4–89.3)	83.0 (77.5–87.5)	0.115
Lymphocyte %	10.7 (8.0–15.7)	9.8 (6.5–15.7)	11.6 (8.0–15.7)	0.073
Platelet, ×10^9^	183 (150–227)	187 (157–232)	181 (146–224)	0.129
CRP	7.1 (2.6–17.3)	17.0 (5.8–30.0)	5.6 (2.1–11.1)	<0.001
SII	1,445	2,260	1,061	<0.001
	(821–2,187)	(1,747–3,218)	(737–1,671)	

*Data are n (%), or median (interquartile)*.

*DCI, delayed cerebral ischemia; ACA, anterior cerebral artery; ICA, internal carotid artery; MCA, middle cerebral artery; VBA, vertebrobasilar artery; RBC, red blood cell; WBC, white blood cell; CRP, c-reactive protein; SII, systemic immune-inflammation index*.

**Table 2:** The univariate and multivariate logistic analysis of predictors for delayed cerebral ischemia.

**Predictors**	**Univariate analysis**		**Multivariate analysis**	
	**OR (95% CI)**	***P*** **-value**	**OR (95% CI)**	***P*** **-value**
Age	1.008 (0.989–1.028)	0.401		
Sex	1.443 (0.897–2.321)	0.131		
Hypertension	0.874 (0.546–1.400)	0.576		
Diabetes	0.923 (0.462–1.844)	0.821		
Hunt-Hess grade	10.073 (5.845–17.360)	<0.001	1.635 (0.678–3.946)	0.274
Modified Fisher grade	17.424 (6.199–48.975)	<0.001	7.851 (2.312–26.661)	**0.001**
Location	1.020 (0.810–1.283)	0.868		
Size	1.174 (0.818–1.684)	0.385		
Clipping	2.470 (1.467–4.160)	0.001	1.685 (0.780–3.639)	0.184
Acute hydrocephalus	5.601 (2.924–10.730)	<0.001	1.689 (0.656–4.346)	0.277
RBC, ×10^9^	0.558 (0.376–0.829)	0.004	0.665 (0.205–2.153)	0.496
Hemoglobin	0.981 (0.969–0.995)	0.006	0.997 (0.961–1.035)	0.886
WBC, ×10^9^	1.286 (1.192–1.387)	<0.001	1.099 (0.979–1.234)	0.109
Neutrophil %	1.018 (0.989–1.049)	0.225		
Lymphocyte %	0.996 (0.992–1.001)	0.076	1.034 (0.984–1.086)	0.189
Platelet, ×10^9^	1.021 (0.986–1.054)	0.236		
CRP	1.025 (1.015–1.036)	<0.001	1.011 (0.998–1.025)	0.109
SII	1.001 (1.001–1.002)	<0.001	1.001 (1.001–1.002)	**<0.001**

*OR, odds ratio; CI, confidence interval*.

*P < 0.05, significant P values are bold*.

**Table 3:** Diagnostic values of SII, NLR, and PLR for delayed cerebral ischemia.

**Variable**	**AUC (95% CI)**	***P*** **-value^a^**
SII	0.860 (0.818–0.896)	Reference
NLR	0.848 (0.805–0.885)	0.3230
PLR	0.745 (0.695–0.791)	<0.001

*NLR, neutrophil to lymphocyte ratio; PLR, platelet to lymphocyte ratio; AUC, area under the curve*.

a *The discriminative performances of SII vs. NLR and SII vs. PLR were compared using the DeLong test*.

Patients were further assigned to two groups based on the SII threshold (Table 4). Patients with an SII level of ≥1,424 had greater modified Fisher and Hunt-Hess scores as well as higher incidence of acute hydrocephalus and DCI. The functional outcome at 3 months was comparatively poorer in patients with SII ≥ 1,424 than those with SII <1,424 [mRS score 3–6: SII ≥ 1,424 = 77/168 (45.8%) vs. SII <1,424 = 15/165 (9.1%); *P* < 0.001]. Furthermore, the risk of DCI was also associated with SII value (SII ≥ 1,424 vs. SII <1,424, OR: 17.793; 95% CI: 6.948–45.564; *P* < 0.001) as a dichotomous variable in the logistic regression model (Table 5).

**Table 4:** Baseline characteristics of included SAH patients dichotomized to the identified SII threshold (1,424).

**Variable**	**SII ≥ 1424**	**SII < 1,424**	***P*** **-value**
	***n*** **= 168**	***n*** **= 165**	
Age (years)	59 (50–66)	59 (53–67)	0.654
Sex (women)	106 (63.1%)	99 (60.0%)	0.562
Hypertension	95 (56.5%)	94 (57.0%)	0.938
Diabetes	23 (13.7%)	22 (13.3%)	0.924
Hunt-Hess grade			<0.001
Grade 1,2,3	92 (54.8%)	143 (86.7%)	
Grade 4,5	76 (45.2%)	22 (13.3%)	
Modified Fisher grade			<0.001
Grade 1,2	31 (18.5%)	70 (42.4%)	
Grade 3,4	137 (81.5%)	95 (57.6%)	
Location			0.504
ACA	60 (35.7%)	55 (33.3%)	
ICA	56 (33.3%)	67 (40.6%)	
MCA	26 (15.5%)	24 (14.5%)	
VBA	26 (15.5%)	19 (11.5%)	
Size (mm)			0.101
<5	91 (54.2%)	102 (61.8%)	
5–10	59 (35.1%)	55 (33.3%)	
>10	18 (10.7%)	8 (4.8%)	
Acute hydrocephalus	40 (23.8%)	8 (4.8%)	<0.001
DCI	94 (56.0%)	7 (4.2%)	<0.001
mRS			<0.001
0–2	91 (54.2%)	150 (90.9%)	
3–6	77 (45.8%)	15 (9.1%)	

*Data are n (%), or median (interquartile)*.

*mRS, modified Rankin scale*.

**Table 5:** Associations of the SII with delayed cerebral ischemia.

	**Univariate analysis**		**Multivariate analysis**	
	**OR (95% CI)**	***P*** **-value**	**OR (95% CI)**	***P*** **-value**
SII^a^	1.001 (1.001–1.002)	<0.001	1.001 (1.001–1.002)	<0.001
SII^b^	28.672 (12.678–68.840)	<0.001	17.793 (6.948–45.564)	<0.001

*Multivariate logistic regression analysis with adjustment for Hunt-Hess grade, modified Fisher grade, treatment methods, acute hydrocephalus, RBC, hemoglobin, WBC, lymphocyte %, and CRP*.

a *SII as a continuous variable*.

b *SII as a dichotomous variable*.

## Discussion

DCI is the leading determinant of poor functional outcomes in patients who have survived after an aneurysmal SAH. We evaluated the SII value for its potential as a novel biomarker for DCI prediction in aneurysmal SAH patients. Our findings indicated that aneurysmal SAH patients with an SII value of ≥1,424 on admission tended to have a worse neurological status and more severe clinical course (e.g., acute hydrocephalus), DCI, and poorer functional outcomes. The SII value on admission was independently associated with the occurrence of DCI. ROC curve analysis demonstrated that SII could predict the occurrence of DCI (AUC = 0.860), suggesting that the SII value on admission may be a promising predictive biomarker for DCI in aneurysmal SAH patients (17).

Aneurysmal SAH is associated with systemic inflammatory response syndrome. Bleeding in the subarachnoid space triggers a rapid activation of the inflammatory cascades, leading to leukocytosis and platelet aggregation (9, 18, 19). Patients with a higher Hunt-Hess grade tended to have obvious alterations in these parameters, indicating that the degree of platelet aggregation and inflammation is positively correlated with early brain injury. Inflammation and thrombotic formation also participate in the pathophysiological process following aneurysmal SAH (18, 20) and can increase the susceptibility to DCI (21). SII is calculated as platelet count × neutrophil count/lymphocyte count, which reflects the balance of a person's inflammatory and immune status. An elevated SII value represents both pro-thrombotic (higher platelet counts) and immune dysregulation (higher neutrophil counts and lower lymphocyte counts) states. Collectively, our study reveals that SII is an independent risk factor for DCI development, which adds to the growing evidence that inflammation and thrombotic formation participate in the pathogenesis of DCI.

Previous studies have shown the clinical significance of SII in hemorrhagic stroke. An increased SII obtained at admission or obtained at 1 day after initial hemorrhage is associated with poor functional outcomes in patients with spontaneous intracerebral hemorrhage (22, 23). However, secondary intracerebral hemorrhage, including SAH, was ruled out in these studies. Yun et al. have performed an analysis for SII in patients with SAH. They found that an elevated SII at admission was an independent risk factor of poor functional outcomes in SAH patients (24). However, it is still unknown whether SII could predict the occurrence of DCI in SAH patients. This study is the first to establish an association between SII and DCI.

Previous research has shown that other inflammatory markers, such as CRP, total leukocyte count, PLR, and NLR, are associated with the development of DCI (10, 25). However, in our study, CRP and total leukocyte count were not independent risk factors for DCI. In the ROC analysis, the AUC for SII was larger than those for NLR and PLR, though the difference was not significant between NLR and SII. SII may be considered as a combination of NLR and PLR because the components of each parameter are encompassed within the SII. When SII was set as a dichotomous variable, we found that the SII value of ≥1,424 was associated with a 17.79-fold increased risk toward DCI, which implied its strong predictive impact. SII is a novel prognostic biomarker in the area of cancer research (11–13). It has several advantages over other inflammation biomarkers. The measurement of SII is affordable, rapid, and widely available in routine laboratory testing. It reflects both the inflammatory and thrombotic status of patients. Additionally, due to the fact that this index is derived from a ratio of two fitted parameters, SII is likely more reliable than other parameters (26).

We postulate here a few reasons why the SII value is elevated in aneurysmal SAH patients with DCI. Firstly, a high SII value can reflect a severe inflammatory response following aneurysmal SAH. Neuro-inflammation may cause disruption in the blood-brain barrier, synaptic injury, neuronal death, white matter injury, and loss of long-term potentiation, all of which ultimately lead to brain injury (27). Several inflammatory molecules and signaling pathways have been found to participate in the development of vasospasm (28). Secondly, an elevated SII value reflects a prothrombotic state and stronger aggregation ability of platelet. Platelet aggregation may induce micro-thrombosis, ischemia, and cerebral tissue death after aneurysmal SAH (29). Thirdly, an elevated SII value also reflects an immune dysregulation state (27, 30). Activation of an innate immune response, which is characterized by increased neutrophil counts, may lead to secondary brain injury after SAH. Microglia cells are part of the brain's innate immune system. Inflammatory reaction following an aneurysmal SAH can trigger microglia cells in the microvasculature, thereby leading to neuronal cell death (31). This evidence suggests that an elevated SII represents a severe inflammation response, a pro-thrombotic state, and an immune dysregulation state, making it easier to understand why an elevated SII value may be associated with the development of DCI. However, more studies are warranted to validate this hypothesis.

The use of transcranial doppler ultrasonography, CT perfusion imaging, and electroencephalography is recommended for monitoring DCI (16). However, compared with these monitoring methods, SII measurement is more convenient and widely available. We postulate that an elevated SII represents a severe inflammation response, a pro-thrombotic state, and an immune dysregulation state. The SII can be used in clinical settings to estimate a patient's susceptibility to DCI and help clinicians recognize patients who are at high risk. It can also guide decisions about which patients should receive prophylaxis against DCI. For example, several studies have shown the safety and efficacy of antiplatelet therapy in SAH (32–34). Antiplatelet drugs with both an anti-platelet and an anti-inflammatory action can reduce their risk of DCI. It may be appropriate to start antiplatelet therapy for patients with higher SII, as these patients are likely to be in a severe inflammation response and a pro-thrombotic state.

Nevertheless, there are some limitations that need to be addressed. Firstly, this was a single-center retrospective observational study. Therefore, it may prone to bias and limit the generalizability of the findings. Secondly, we did not assess the changes in SII values over time after hospital admission. Thirdly, the definition of DCI, especially as applied to patients who are sedated or comatose, may exclude patients who have actually developed DCI. Fourthly, we did not explore the relationship between SII and functional outcomes in patients with aneurysmal SAH. Clinical observational studies may overestimate the causality between the SII and DCI, and a prospective, multi-center study is needed to verify our findings in the near future.

## Conclusion

SII is an independent predictor of DCI in patients with aneurysmal SAH. The SII value of ≥1,424 on admission may guide clinicians to screen for patients who are at high risk for DCI.

## Data Availability Statement

The raw data supporting the conclusions of this article will be made available by the authors, without undue reservation.

## Ethics Statement

The studies involving human participants were reviewed and approved by The Ethics Committee of the Shanghai Tenth People's Hospital. The patients/participants provided their written informed consent to participate in this study.

## Author Contributions

QZ: conception, design, and administrative support. LC and SP: provision of study materials or patients. SP: collection and assembly of data. RS and YX: data analysis and interpretation. All authors manuscript writing and final approval of manuscript.

## Conflict of Interest

The authors declare that the research was conducted in the absence of any commercial or financial relationships that could be construed as a potential conflict of interest.

## Publisher's Note

All claims expressed in this article are solely those of the authors and do not necessarily represent those of their affiliated organizations, or those of the publisher, the editors and the reviewers. Any product that may be evaluated in this article, or claim that may be made by its manufacturer, is not guaranteed or endorsed by the publisher.
